# Association of Lung Ultrasound Scores With Different Modes of Respiratory Support and Clinical Outcomes: An Observational Study in a Tertiary Care Neonatal Unit

**DOI:** 10.7759/cureus.66199

**Published:** 2024-08-05

**Authors:** Keshav Kumar Pathak, Arti Maria, Munish Guleria, Pranaya Kumar Mall, Abhinav Sharma

**Affiliations:** 1 Department of Neonatology, All India Institute of Medical Sciences, Patna, Patna, IND; 2 Department of Neonatology, Atal Bihari Vajpayee Institute of Medical Sciences and Dr. Ram Manohar Lohia Hospital, New Delhi, IND; 3 Department of Radiodiagnosis, Atal Bihari Vajpayee Institute of Medical Sciences and Dr. Ram Manohar Lohia Hospital, New Delhi, IND

**Keywords:** lung ultrasound score, neonate, clinical outcome, association, respiratory support

## Abstract

Background: Lung ultrasound (LUS) is an evolving point-of-care tool in the neonatal intensive care unit. LUS score has been evaluated in adults as well as in neonates to characterize and diagnose various respiratory conditions. Recently, the LUS score has been evaluated for predicting clinical respiratory outcomes in neonates.

Objective: To assess the association between LUS score and various modes of respiratory support and clinical outcomes among neonates presenting with respiratory distress.

Methods: In this prospective, cross-sectional, observational study done in a tertiary care neonatal unit, the LUS score was calculated within three hours of receiving respiratory support. Subsequently, the LUS score was assigned with each escalation and de-escalation of respiratory support. Maximum LUS scores for each clinical outcome were also recorded. Inter-rater agreement was determined with the intraclass correlation coefficient.

Result: A total of 162 LUS scans were performed in 65 babies with a mean gestation of 32.4 ± 3.7 weeks and median (IQR) birth weight of 1480 (1130-2000) grams. The LUS scores (median (IQR)) of babies on continuous positive airway pressure (CPAP), noninvasive positive pressure ventilation (NIPPV), and mechanical ventilation (MV) were 4 (3-6.5), 9 (8-11), and 12 (11-13.5), respectively (p-value < 0.001). The difference in maximum median LUS scores between different clinical outcomes was statistically significant, with a p-value < 0.001. LUS score had an excellent inter-rater agreement (intraclass correlation coefficient = 0.998; p-value < 0.001).

Conclusion: There is an association between LUS score and different modes of respiratory support with scores increasing as the level of support increased. LUS score was also found to be related with clinical outcomes like death, extubation failure, and recovery, which could help in predicting the prognosis.

## Introduction

Respiratory distress in neonates is one of the most common reasons for admission to the neonatal intensive care unit (NICU) [1]. Neonates may require different levels of respiratory support based on the severity of respiratory distress like continuous positive airway pressure (CPAP), noninvasive positive pressure ventilation (NIPPV), and mechanical ventilation (MV). There are various guidelines to aid in this decision-making, most of which are semi-objective, aided by chest X-ray and blood gas analysis, or based on clinical judgment [2-4]. There is a felt need for more objective tools that may help in the early optimization of respiratory support in neonates. Lung ultrasound (LUS) is one such evolving point-of-care tool that is inexpensive, easily accessible, radiation-free, and is being used increasingly in NICUs now [5]. Studies have used LUS scores in adults, which are also applicable in neonates with some modification and have been validated in this population [6]. Based on artifacts seen on LUS, characteristic and specific LUS patterns have been described for various respiratory morbidities [7]. However, most of the studies on neonates have utilized LUS mainly for diagnostic purposes. Recently, the LUS score has also been evaluated for predicting clinical respiratory outcomes like the need for mechanical ventilation, the need for surfactant, and successful extubation [8,9]. LUS score is inversely correlated with lung aeration. We hypothesized that LUS scores change with the severity of distress and the level of respiratory support required [10]. If so, this may serve as a tool to objectively decide on the required level of respiratory support and to track disease evolution. Prediction models may later be developed to decide the optimal level of respiratory support based on LUS scores. With this background, we conducted this observational study in neonates with respiratory distress to evaluate the change in LUS scores with various modes of respiratory support. The secondary aim was to assess the association between LUS scores and various clinical outcomes.

## Materials and methods

This study was a cross-sectional observational study conducted in a 30-bed level III NICU in a tertiary care center in North India with an annual admission of approximately 700 between August 2020 and September 2021.

The primary objective of the study was to assess the association of LUS scores with various modes of respiratory support among neonates presenting with respiratory distress. The secondary objective was to assess the association of LUS score with various clinical outcomes like recovery, extubation failure, and death. Ethical clearance for the protocol was obtained from the institutional ethical committee.

All neonates admitted to the NICU were assessed for eligibility by applying inclusion and exclusion criteria. Once eligible, informed verbal and written consent in the local language was obtained from parents before enrolment. Neonates presenting with respiratory distress having Downes or Silverman scores of more than or equal to 3 were included in the study. Any baby with apnea or gasping due to respiratory cause was also included. Neonates with gross congenital malformation, respiratory distress due to obvious non-respiratory etiology, and refusal to consent for participation were excluded from the study. All efforts were taken to ensure that babies with non-respiratory pathology were identified early and not included.

Respiratory distress was defined as the presence of any two of the following features: (1) respiratory rate (RR) > 60/minute; (2) subcostal /intercostal recessions; (3) expiratory grunting/groaning. Respiratory support included CPAP, NIPPV, and invasive mechanical ventilation (IMV). Clinical outcomes were categorized into the following three types: (1) recovery was defined as not requiring any respiratory support for seven days after weaning off; (2) extubation failure was labeled if the baby required reintubation within three days of extubation; (3) death on respiratory support or within three days of discontinuation of respiratory support was attributed to being a result of respiratory morbidity.

Babies were provided respiratory support, surfactant, and other supportive treatment by a person other than the principal investigator (PI), according to our standard unit protocol based on the European Consensus Guidelines on the Management of Respiratory Distress Syndrome 2019 Update [3]. Demographic details, clinical data, respiratory distress score, and respiratory support variables of enrolled babies were recorded in a predesigned case proforma.

Lung ultrasound

Newborns meeting the enrollment criteria were subjected to an initial LUS scan within three hours of receiving any respiratory support. LUS score was calculated and assigned for that respiratory support. Subsequently, for all enrolled babies, with each escalation or de-escalation of respiratory support, LUS was repeated within three hours of a change in support, and LUS score was assigned for that particular level of support. LUS scores were also calculated when babies were de-escalated from respiratory support to room air. However, considering feasibility and logistic issues, a minimum gap of six hours was kept between two consecutive LUS scans. If any change in respiratory support occurred within six hours, then the LUS score at the highest level of respiratory support within that period was recorded. This process was continued till the baby either recovered as defined or attained the outcome of death. LUS scans were performed by the PI after receiving formal training from a senior ultrasonologist for a duration of two months. At the end of the training session, the senior ultrasonologist validated serial multiple LUS scans done by the PI for establishing inter-rater agreement. The PI was considered trained only when the agreement of accuracy between the senior ultrasonologist and the PI was more than 80%. After the completion of the training session, LUS scans were performed and scored independently by the PI, who also stored the soft copies of LUS images in a USB drive for the final assignment of LUS scores by the senior ultrasonologist. While assigning the final LUS score, the senior ultrasonologist was kept blinded to the baby’s clinical state. To maintain uniformity and quality, if PI was not available due to any reason, eligible babies during that period were not enrolled in the study.

Lung ultrasound procedure

**Figure 1:**
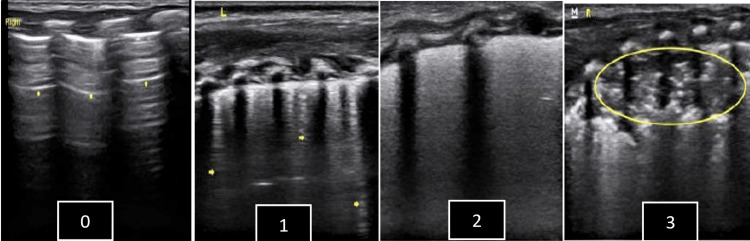
Description of the lung ultrasonography score. Scores are given as follows for any lung area: 0 indicates A-pattern (defined by the presence of only A-lines, arrow); 1 indicates B-pattern (defined as the presence of ≥ three B-lines, arrow, well-spaced); 2 indicates severe B-pattern (defined as the presence of crowded and coalescent B lines with or without consolidations limited to subpleural space); and 3 indicates extended consolidation (circle).

A Sonosite M-Turbo USG machine (FUJIFILM Sonosite, Inc., Bothell, WA) with a high frequency (6-13 MHz) linear probe was used for the LUS image. For calculating the LUS score, each lung was divided into three areas by the anterior and posterior axillary line into the anterior, posterior, and lateral areas. A total of six areas were scanned during the calculation of LUS. Babies were kept supine to obtain an image of the anterior and lateral areas, whereas the image of the posterior area was taken in the lateral or prone position. For taking the image, the probe was placed in a perpendicular/vertical plane. The average time taken for doing LUS was three to five minutes and examinations were well tolerated by babies. The LUS score used in the present study was modified from an index proposed for adult patients [11]. For each lung area, a 0-to-3-point score was given (total score ranging from 0 to 18) as follows: 0 - "A pattern" defined by the presence of A-lines only or presence of < three B lines; 1 - "B pattern" defined as the presence of ≥ three well-spaced B lines; 2 - "Severe B pattern" defined as the presence of crowded or coalescent B lines with or without consolidation limited to the subpleural space; 3 - extended consolidation with air bronchogram. Figure 1 summarizes the LUS score characteristics.

Sample size calculation

Hitherto before, at the time when the present study was conceived, LUS had neither been evaluated for assessing the type of respiratory support required nor for tracking disease outcomes among neonates with respiratory distress. Due to a lack of baseline data, we decided to enroll babies such that at least 30 LUS scans may be analyzed for each level of respiratory support.

Statistical analysis

To compare the outcome variables between inter-related groups, a continuous scale nonparametric test (Friedman two-way ANOVA) was used. To compare the outcome variable between independent samples, the Mann-Whitney U test and the Kruskal-Wallis test were used as appropriate. To compare the baseline and outcome variables on nominal type of data, the chi-square test or Fisher's exact test was used as appropriate. A two-sided p-value < 0.05 was considered significant. To compare the median LUS score between respiratory support groups, a p-value < 0.01 was considered significant. The intraclass correlation coefficient was used to see the agreement between the PI's LUS score and the senior ultrasonologist's LUS score. Statistical analysis was performed using SPSS Statistics version 20.0 for Windows (IBM Corp., Armonk, NY).

## Results

**Figure 2:**
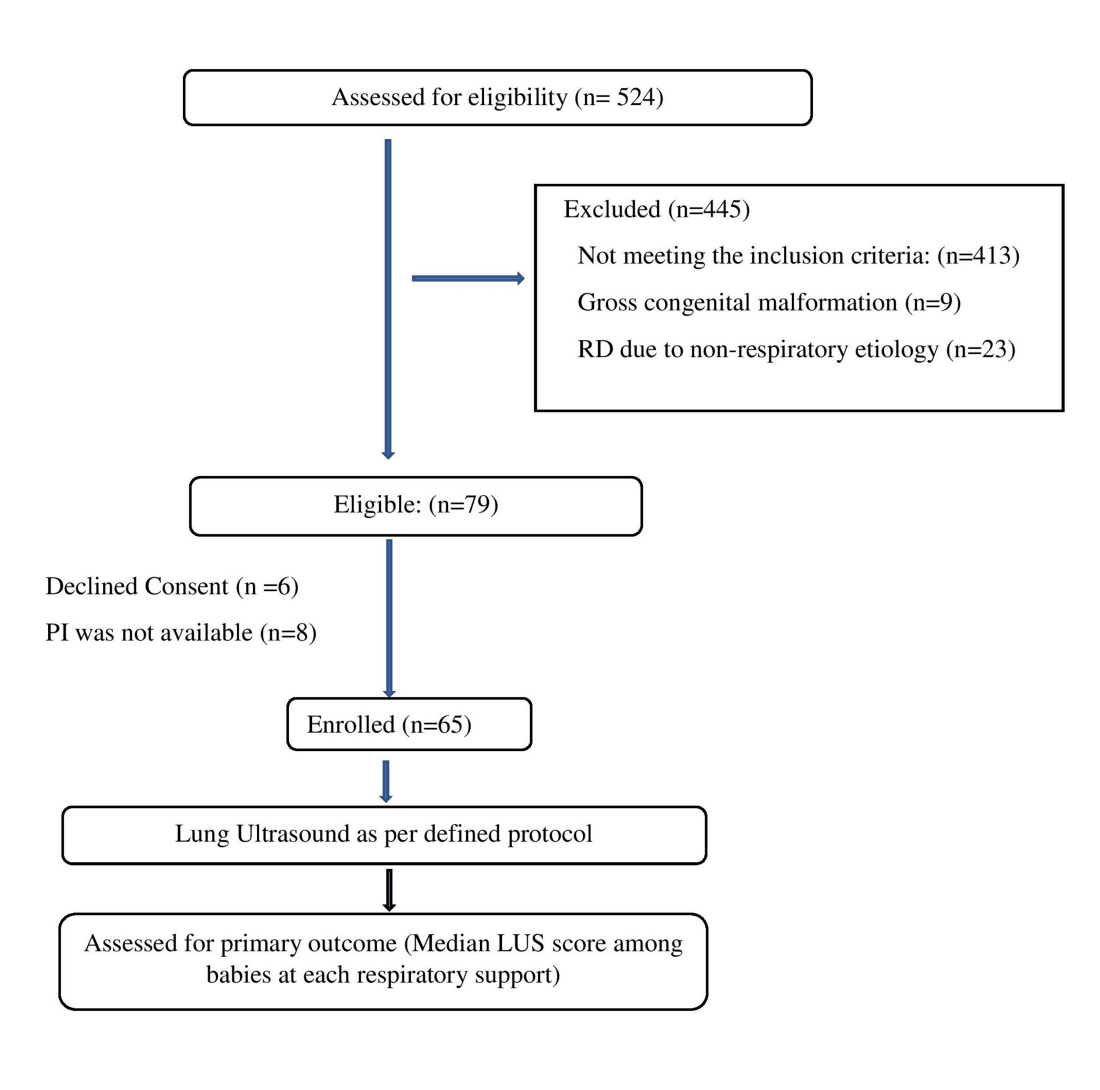
Study flowchart. RD: respiratory distress; PI: principal investigator; LUS: lung ultrasound.

**Figure 3:**
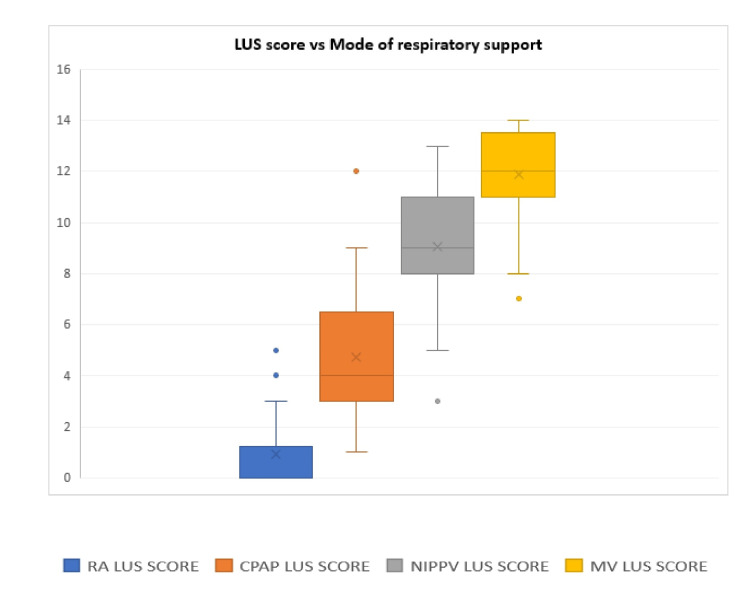
LUS score vs. mode of respiratory support. LUS: lung ultrasound; CPAP: continuous positive airway pressure; NIPPV: noninvasive positive pressure ventilation; MV: mechanical ventilation; RA: room air.

During the study period, 524 babies were assessed for eligibility. Out of these, 413 babies did not meet the inclusion criteria, and 32 babies were excluded (nine babies had gross congenital malformation and 23 babies had nonrespiratory etiology for respiratory distress). Out of 79 eligible babies, six babies were not enrolled as parents refused to participate and eight babies were not enrolled as the PI was not available. Finally, 65 babies were enrolled in the study (Figure 2). A total of 162 LUS scans were performed during the study period. Out of a total of 162 LUS scans, 54 (33%) were performed on babies on room air, 57 (35%) were done while babies were on CPAP support, 26 (16%) were done on NIPPV support, and 25 (16%) were done on MV support. The mean gestational age (weeks) of enrolled babies was 32.4 ± 3.7, and 63.1% were males. Median birth weight (gram) was 1480 (1130-2000). A total of 30.8% of babies were born by cesarean section (Table 1). For the primary outcome, the LUS score increased with increasing modes of respiratory support. Babies on room air had a median (IQR) LUS of 0 (0-1.25). LUS scores (median (IQR)) of babies on CPAP, NIPPV, and MV were 4 (3-6.5), 9 (8-11), and 12 (11-13.5), respectively. The difference was statistically significant with p-value < 0.001 (Table 2 and Figure 3). The median LUS scores between groups (CPAP & NIPPV, CPAP & MV, and NIPPV & MV) were also significant with each group having a p-value < 0.001. For secondary outcomes of association of LUS score with clinical outcomes, the maximum median LUS score for the recovery group was 5.00 (3.00-8.00), extubation failure group was 11.00 (11.00-13.00), and the death group was 12.00 (12.00-13.75). The difference was statistically significant (p-value < 0.001). There was a very high agreement between the PI and senior ultrasonologist in the assignment of the LUS score with an intraclass correlation coefficient of 0.998 with a p-value < 0.001.

**Table 1:** Baseline characteristics. SD: standard deviation; N: number; IQR: interquartile range; LUS: lung ultrasound.

Variables	Result (N = 65)
Gestational age (weeks), mean ± SD	32.4 ± 3.7
Gestational age categories, n (%)	
Extreme preterm	3 (4.6%)
Very preterm	27 (41.5%)
Moderate preterm	12 (18.5%)
Late preterm	10 (15.4%)
Gender, N (%)	
Male	41 (63.1%)
Female	24 (36.9%)
Birth weight in grams, median (IQR)	1480 (1130-2000)
Birth weight categories, n (%)	
<1000 gram	8 (12.3%)
1000-1499 gram	25 (38.5%)
1500 to 2499 gram	21 (32.3%)
≥2500 gram	11 (16.9%)
Enrolment age at first LUS scan (days), median (IQR)	1 (1-2)
Mode of delivery, n (%)	
Vaginal	45 (69.2%)
Cesarean section	20 (30.8%)
Antenatal steroids, n (%)	
Yes	28 (43.1%)
No	20 (30.8%)
Not eligible	17 (26.1%)
Respiratory morbidities at admission, n (%)	
Respiratory distress syndrome	30 (46.1%)
Transient tachypnea of the newborn	20 (30.8%)
Pneumonia	14 (21.5%)
Meconium aspiration syndrome	1 (1.6%)

**Table 2:** Primary outcome: difference in median LUS score at different levels of respiratory support. LUS: lung ultrasound; CPAP: continuous positive airway pressure; NIPPV: noninvasive positive pressure ventilation; MV: mechanical ventilation.

Level of respiratory support	Median LUS score (IQR)	P-value
Room air	0 (0-1.25)	<0.001
CPAP	4.00 (3.00-6.50)	
NIPPV	9.00 (8.00-11.00)	
MV	12.00 (11.00-13.50)	

## Discussion

The present cross-sectional observational study was conceptualized and conducted in neonates with respiratory distress to provide preliminary baseline information regarding the evolution of LUS scores, if any, at various levels of respiratory support. We included babies across all gestations and with varied respiratory morbidities. Although we had not put any gestational age cut-off, most of the babies (41%) in our study were very preterm. This was possibly because respiratory distress is epidemiologically and biologically more common in preterm babies. The results may not be generalized and extrapolated for late preterm and term babies. Of the enrolled babies, 46% had respiratory distress syndrome as the primary diagnosis, 31% had transient tachypnea of the newborn, 22% had pneumonia, and 1% had meconium aspiration syndrome (MAS). Thus, our study included babies with diverse clinical spectrums, by virtue of which the LUS score may be regarded useful, especially in babies with respiratory distress syndrome, transient tachypnea of the newborn, and pneumonia. However, as we had very few babies with MAS, the study results may not be consistent in babies with this particular morbidity. This is especially relevant as previous studies have reported some dissociation between clinical severity and imaging findings in babies with MAS [12].

In our study, there was a statistically significant difference in the median (IQR) LUS score of babies on room air, CPAP, NIPPV, and invasive ventilation. We acknowledge the fact that the sample size was taken arbitrarily due to a lack of previous baseline data and therefore, further adequately powered studies are warranted to validate these findings. To date, there are hardly any studies that have evaluated the evolution of LUS scores at various levels of respiratory support in babies with diverse clinical spectrums. A similar study by Szymański et al. evaluated the association of serial LUS scores with different ventilation modes, but the study only included babies less than 32 weeks with respiratory distress syndrome. They also reported a linear increase in LUS score with the escalation of respiratory support, as we have found in our study [13]. Very recently, Raimondi et al. investigated the utility of LUS as a bedside tool to monitor respiratory status in neonates [14]. This study also included only preterm babies. It is worthwhile to mention that both these studies used oxygen saturation/fraction of inspired oxygen (SpO2/FiO2) as a marker of oxygenation status and disease evolution and showed a significant positive correlation between the LUS scores and the SpO2/FiO2 ratio. We acknowledge that we did not correlate the LUS scores with any objective index for respiratory distress.

Another noteworthy point is that, in our study, we used a modified approach for calculating the LUS score. We assessed anterior, lateral, and posterior lung fields, unlike the majority of the previous studies that have described the anterior-lateral approach largely. Clinical observations have shown that pulmonary pathology in neonates often tends to be located in the posterior lung field, possibly because of gravitational effect [15-17]. Therefore, in neonates, overlooking the posterior lung field may increase the chances of missing important findings and accordingly affect the LUS scores. This was demonstrated by Szymański et al., who included the posterior lung field in their LUS scan. In that study, the posterior pulmonary field scores were significantly higher compared to anterior lung field scores [13].

Other than this, studies by Raimondi et al. also analyzed LUS scans based on anterior and lateral lung fields [18,19]. The difference between the maximum median LUS score for the different clinical outcomes was also statistically significant. Previous studies have also evaluated the utility of LUS scores in predicting outcomes like the need for mechanical ventilation and extubation failure and reported similar observations. A study done by Gunes et al. reported a positive correlation between LUS scores (at two and six hours of life) and positive end-expiratory pressure (PEEP) value, need for mechanical ventilation, total mechanical ventilation days, length of hospital days, and antibiotic days [20]. Various authors have also evaluated the diagnostic ability of LUS scores to predict weaning success or extubation failure in ventilated neonates and have reported that LUS scores at specific cut-offs and time points could predict extubation failure [21-23]. We understand that LUS is operator-dependent and this may limit the replicability of the study. However, despite being operator-dependent, performing the LUS procedure is easy to master and has a steep learning curve. Based on this study and studies done by various authors, we can comment that the LUS examination can be easily learned over one to two months of supervised training and practice. In this present study, the LUS score assignment had a very high inter-rater agreement between the principal investigator and senior ultrasonologist, as reflected by a very high intraclass correlation coefficient of 0.998 with a p-value < 0.001. Szymański et al. also showed a very high intraclass correlation coefficient of 0.94 and Cronbach’s alpha (0.99), showing excellent inter-rater agreement [13]. A study done by Raimondi et al. also showed full interobserver agreement in the image interpretation to categorize LUS scans into different categories with қ = 1 [19]. The study by Brat et al. also showed very high interobserver agreement (қ = 0.89) between the resident physician and senior neonatologist expert in LUS for image interpretation [11]. Thus, once formal training is acquired, LUS has good replicability.

Strengths of the study

The concept is novel, and hitherto, there is very limited evidence on the change and evolution of LUS scores at various levels of respiratory support. To the best of our knowledge, this is the first study to evaluate and report changes in LUS scores at different levels of respiratory support in such a diverse gestational age group and respiratory conditions. Babies requiring respiratory support were managed according to the uniform unit protocol, thereby increasing the internal validity of this study. In maximum studies on the LUS score, anterior and lateral lung fields were included, but in this study, posterior lung field was also included, which makes the study clinically more robust as many respiratory pathologies in neonates tend to have gravitational positioning.

Limitations of the study

We acknowledge the following limitations in our study. The sample size of the study was small. To relate the results of this cross-sectional observational study to the general population, a bigger sample size is required with a long duration of the study period. The results may not be generalizable to late preterm, term babies, and babies with MAS, as their numbers were small. The replicability of the study is limited in centers where trained personnel for performing LUS is not available, which may be the case in many resource-limited settings.

## Conclusions

There is an association between LUS scores and the level of respiratory support. There was a significant difference in median LUS scores at various levels of respiratory support, with scores increasing as the level of support increased. The results need to be validated further in adequately powered studies with large sample sizes, which may help in establishing LUS as a bedside tool to monitor respiratory status in neonates with respiratory distress and timely optimize respiratory support accordingly.
